# Blood transfusion, colloid therapy and the possible saving of albumin volumes during surgery: data analysis of the survey for certified hospitals of the Japanese Society of Anesthesiologists

**DOI:** 10.1007/s00540-015-2136-3

**Published:** 2016-01-14

**Authors:** Hideki Miyao, Safety Committee of Japanese Society of Anesthesiologists

**Affiliations:** Department of Anesthesiology, Saitama Medical Center, Saitama Medical University, 1981, Kamoda, Kawagoe, Saitama 350-8550 Japan; Japanese Society of Anesthesiologists, 1-5-2, Minami-cho, Minatojima, Chuo-ku, Kobe, 650-0047 Japan

**Keywords:** Transfusion, Volume therapy, Hydroxyethyl starch, Albumin

## Abstract

**Purpose:**

Third-generation hydroxyethyl starch (HES) 130/0.4 has a larger dose limitation (up to 50 mL/kg/day) than HES 70/0.5 (up to 1000 mL/day) which has been used in Japan for 40 years. The aim of this study was to survey the current intraoperative blood transfusion and volume therapy and to predict the possible reduction of intraoperative albumin consumption assuming further replacement by HES 130/0.4 using data obtained from a survey by the Japanese Society of Anesthesiologists (JSA), although HES130/0.4 was not launched in Japan during this survey period.

**Methods:**

In a JSA survey conducted at JSA-certified hospitals, 12,856 patients with a certain amount of blood loss were analyzed for 1 month (April, 2012). The patients were divided into two groups—group A included patients aged ≥11 years and group B included patients aged <10 years. The possible lower volume of intraoperative albumin was calculated assuming that HES 130/0.4 was used up to a dose of 50 mL/kg.

**Results:**

Blood loss (total 15,111 L; 15,057 L in group A and 54 L in group B) was treated with allogeneic transfusion (total 7970 L; 7893 L in group A and 77 L in group B) and auto-transfusion (total 1777 L; 1771 L in group A and 6 L in group B) in both groups (*n* = 11,670 and 119). Albumin (total 1391 L; 1376 L in group A and 15 L in group B), and HES 70/0.5 (total 7645 L; 7638 L in group A and 7 L in group B) were used in both groups (*n* = 10,850 and 116). Five percent and 4.4 % albumin (total 1189 L; 1180 L in group A and 9 L in group B) could be replaced by HES 130/0.4 if HES 130/0.4 had been used up to a dose of 50 mL/kg.

**Conclusion:**

Blood loss (15,111 L) was replaced with allogeneic transfusion (53 %), auto-transfusion (12 %), albumin (9 %) and HES 70/0.5 (51 %) during surgery in April 2012. The predicted volume of 5 and 4.4 % albumin saved during this 1-month period if HES 130/0.4 had been used up to a dose of 50 mL/kg was 1189 L (86 % of actual amount used).

## Introduction

Perioperative fluid therapy has shifted from the liberal use of crystalloids to goal-directed volume-restricted therapy using colloids for blood loss substitution [1–3]. In Japan, hydroxyethyl starch (HES) is the preferred colloid during surgery with HES 70/0.5 (Hespander^®^/Salinhes^®^) being the only HES specification available for the past 40 years. The maximum daily dosage of HES 70/0.5 has been limited to 1000 mL. When a higher volume of a colloid solution was indicated, albumin had to be used instead of HES 70/0.5 because new generation of HES had not been available in Japan, resulting in increased consumption of intraoperative albumin. Third-generation HES 130/0.4 (Voluven^®^) has a higher dose limitation (50 mL/kg/day) than HES 70/0.5 and was launched in Japan in October 2013.

In July 2012, 1 year prior to the launch of HES 130/0.4, the Japanese Society of Anesthesiologists (JSA) initiated a survey for usage of albumin and HES 70/0.5 within JSA-certified hospitals to document intraoperative infusion and transfusion therapy including colloid therapy (HES, albumin, and others). The aim of this study was to investigate the current status of blood transfusion and colloid therapy in operating theaters in Japan and to predict the possible reduction of intraoperative albumin volumes assuming that colloid volumes up to 50 mL/kg could be replaced by HES 130/0.4.

## Methods

After approval by the ethics committee of the JSA, questionnaires prepared by the Safety Committee of the JSA were sent to 1234 JSA-certified hospitals in 2012. The survey consisted of two parts. In the first part, the representatives of anesthesiologists at individual hospitals were asked to complete a questionnaire on the use of 6 % HES 70/0.5 and albumin in the operating theater. In the second part, data describing transfusion and infusion status of surgical patients with intraoperative blood loss of either ≥500 mL for patients aged ≥11 years (group A) or with intraoperative blood loss ≥10 mL/kg for patients aged <10 years (group B) was derived from a 1-month database (April 2012).

The database from the second part of the JSA survey 2012 was the subject of the present study. The results of the first part will be presented soon on the official home page of JSA.

Unknown blood loss from certain procedures such as cardiac surgery with cardio-pulmonary bypass and abdominal aortic aneurysm surgery with intraoperative autologous blood salvage was not counted in the analysis of blood loss. Blood loss reported including amniotic fluid in Caesarian section or ascites in abdominal surgery was adopted to the blood loss as they were. The amount of transfusion was documented in mL, but when units were used, it was calculated as 140 mL per unit of red blood cells or 120 mL per unit of fresh frozen plasma in a standardized way. Platelet transfusion was excluded from further analysis as the documentation could not be clarified (units or mL) in many cases.

**Fig. 1:**
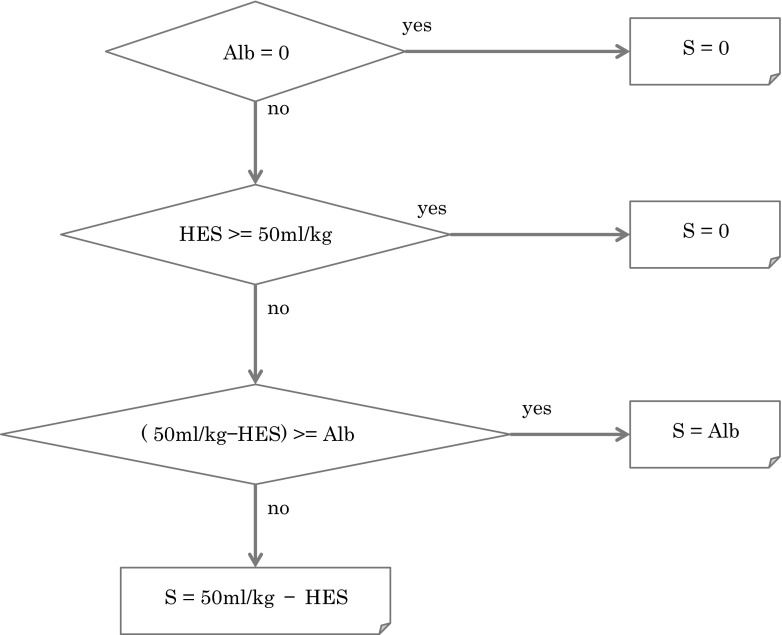
Algorithm for calculation of possible saving of albumin if 6 % HES 130/0.4 had been used up to a total dose of 50 mL/kg instead of albumin. *Alb* intraoperative infusion volume of 5 or 4.4 % albumin, *HES* 6 % HES 70/0.5, *S* saving volume of albumin. Example: If body weight is 60 kg and 5 % albumin (1500 mL) would be used, 1st branch of upper Fig. 1 is "no". If HES (1000 mL) would be used, the 2nd branch is "no" because HES (1000 mL) is smaller than 50 mL/kg (3000 mL/60 kg). The 3rd branch is "yes" because 50 mL/kg (3000 mL)—HES 1000 mL = 2000 mL which is larger than albumin used (1500 mL). This means that HES 130/0.4 could be used up to 3000 mL and this amount is larger than HES 70/0.5 (1000 mL) plus albumin (1500 mL) used. Then, 1500 mL of albumin used could be replaced with HES 130/0.4, consequently saving volume of albumin is whole amount of albumin used (1500 mL). If 5 % albumin (2500 mL) would be used in the upper example, the 3rd branch is "no" because 50 mL/kg (3000 mL)—HES 1000 mL = 2000 mL which is smaller than albumin used (2500 mL). Then, 50 mL/kg (3000 mL)—HES 1000 mL = 2000 mL which is 80 % of albumin used (2500 mL) is the saving volume of albumin

The algorithm to calculate the volume of 5 and 4.4 % albumin that could have been saved if 6 % HES 130/0.4 was used up to a dose of 50 mL/kg is given in Fig. 1. The intraoperative infusion volumes of 20 and 25 % albumin were excluded from the above calculations because these solutions were mostly used in the priming solution of cardiopulmonary bypass circuit.

### Statistics

All variables were analyzed by descriptive statistical analysis.

## Results

**Fig. 2:**
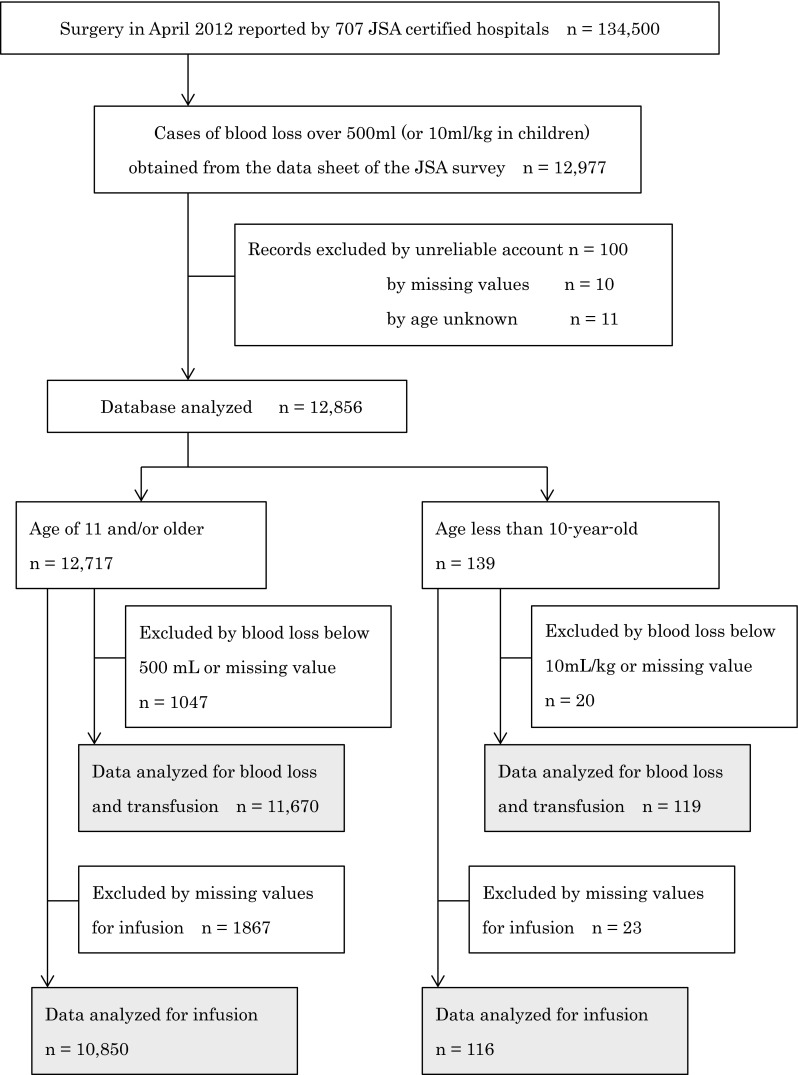
Diagram for data selection

Seven hundred and seven of 1234 JSA-certified hospitals returned valid questionnaires for analysis and reported 134,500 surgical patients of whom 12,977 matched the methodology of this survey. Finally 11,670 patients were suitable for analysis for blood loss/transfusion, with 10,850 patients being suitable for infusion therapy in group A, and 119 patients being suitable for blood loss/transfusion and 116 for infusion therapy in group B (Fig. 2).

Table 1 shows demographic data in groups A and B. Table 2 shows blood loss, allogeneic transfusion, and auto-transfusion of patients in groups A and B. Total blood loss in group A (15,057 L) was treated with allogeneic transfusion (7893 L: 52 % of total blood loss) and auto-transfusion (1771 L:12 % of total blood loss), whereas total blood loss in group B (54,461 mL) was treated with allogeneic transfusion (77,016 mL: 141% of total blood loss) and auto-transfusion (6338 mL: 12 % of total blood loss) . It was evident that transfusion therapy dominates in pediatric patients.

**Table 1:** Demographic data in each group

	Group A	Group B
Age (years)		
Valid cases	11,670 cases	119 cases
Mean (median: min–max)	57 years (62: 11–104)	1.8 years (0.9: 0–10)
Body weight (kg)		
Valid cases	11, 446 cases	119 cases
Mean (median: min–max)	60.2 kg (59.6: 30.1–138.7)	9.7 kg (7.7: 1.7–36.1)

*min* minimum, *max* maximum

**Table 2:** Blood loss, allogeneic transfusion, and auto-transfusion

	Group A	Group B
Blood loss		
Valid cases	11,670 cases	119 cases
Total amount	15,057 L	54,461 mL
Mean (median: min–max)	1292 mL (911: 500–44,950)	458 mL(59) [300 (37): 17 (10)–2890 (389)]
Allogeneic transfusion		
Valid cases	5634 cases	104 cases
Total amount	7893 L	77,016 mL
Mean (median: min–max)	1401 mL (800: 1–48,300)	741 mL (367: 27–5958)
Auto-transfusion		
Valid cases	2624 cases	32 cases
Total amount	1771 L	6338 mL
Mean (median: min–max)	675 mL (500: 1–15,500)	198 mL (148: 4–1030)

Valid cases of blood loss indicates blood loss ≥500 mL in group A and 10 mL/kg in group B. Valid cases of allogeneic and auto transfusion indicates over 0 mL. Allogeneic transfusion included red blood cell concentrate and fresh frozen plasma but not platelet concentrate. Auto-transfusion included preoperative donated autologous transfusion, intraoperative hemodilution autologous transfusion, and intraoperative blood cell salvage transfusion, *min* minimum; *max* maximum, () in group B: values in mL/kg

Table 3 shows the total infusion volume including crystalloids, colloids, and predicted volume of albumin saved in groups A and B. Of 10,850 patients in group A, 1979 (18 %) were given albumin (5, 4.4, 20, or 25 %), and 8464 (78 %) were given HES 70/0.5. Of 8464 patients given HES 70/0.5, 1518 (18 %) were given >1000 mL HES 70/0.5 (this is the limitation volume specified in the package insert). One thousand one hundred eighty liters amount of 5 and 4.4 % albumin, which was 86 % of actually amount used (1366.2 L), could be replaced by HES 130/0.4 if HES 130/0.4 had been used up to a dose of 50 mL/kg. Of 116 patients in group B, 76 (66 %) were given albumin (5, 20, or 25 %), and 45 (39 %) were given HES 70/0.5. It became evident that the rate of albumin given was higher and the rate of HES given was lower in pediatric patients (group B) than those in group A. Nine thousand one hundred seventy four mL amout of 5 % albumin, which was 68 % of actually amount used (13,446 mL), could be replaced by HES 130/0.4 if HES 130/0.4 had been used up to a dose of 50 mL/kg.

**Table 3:** Total infusion volume, colloids volume, and predicted volume of albumin saved

	Group A	Group B
Total infusion volume		
Valid cases	10,850 case	116 cases
Total amount	32,283.3 L	87,431 mL
Mean (median: min–max)	2975 mL (2500: 50–42,940)	754 mL (361: 17–6940)
5 % albumin		
Valid cases	1751 cases	53 cases
Total amount	1280.0 L	13,446 mL
Mean (median: min–max)	731 mL (500: 4–9500)	254 mL (110: 20–1650)
4.4 % albumin		
Valid cases	133 cases	0 case
Total amount	86.2 L	–
Mean (median: min–max)	648 mL (500: 100–3500)	–
20 % albumin		
Valid cases	15 cases	2 cases
Total amount	2.5 L	16 mL
Mean (median: min–max)	163 mL (100: 50–1000)	8 mL (8: 8–8)
25 % albumin		
Valid cases	80 cases	21 cases
Total amount	7.0 L	1640 mL
Mean (median: min–max)	88 mL (100: 30–250)	78 mL (80: 50–130)
HES70/0.5		
Valid cases	8464 cases	45 cases
Total amount	7637.5 L	7131 mL
Mean (median: min–max)	902 mL (,000: 10–12,500)	159 (12) mL [62 (8): 10 (1)–1000 (61)]
Other artificial colloids		
Valid cases	295 cases	0 case
Total amount	195.5 L	–
Mean (median: min–max)	663 mL (500: 50–2000)	–
Albumin saved by HES130/0.4		
Valid cases	1817 cases	52 cases
Total amount	1180.1 L	9174 mL
Mean (median: min–max)	650 mL (500: 4–3350)	176 mL (100: 20–905)

Total infusion volume included crystalloids and colloids. Other artificial colloid was only dextran. 20 and 25 % albumin were ineligible for analysis of "Albumin saved by HES 130/0.4"; *min* minimum, *max* maximum, () in group B: values in mL/kg

The calculation of the total predicted volume of albumin saved for 5 and 4.4 % albumin in groups A and B was 1189 L in April 2012. This predicted volume of albumin saved (1189 L) corresponds to 86.2% of actual amount of 4.4 and 5% albumin (1379 L) used during this period.

The JSA survey 2012 revealed that 15,111 L of blood loss were replaced with 7970 L of allogeneic transfusion, 1777 L of auto-transfusion, 1391 L of albumin, and 7645 L of HES 70/0.5 during surgery in April 2012. The predicted volume of 5 and 4.4 % albumin saved was 1189 L which was 86 % of actual amount used during this 1-month period if HES 130/0.4 had been used up to a dose of 50 mL/kg.

## Discussion

The present survey is a unique study of a large database obtained from 707 JSA-certified hospitals to describe the current blood loss/transfusion and colloid therapy during surgery in Japan. The results of this survey may lead to a new strategy for intraoperative volume therapy.

HES has been used for perioperative volume replacement as a plasma substitute in operating theaters and intensive care units. We should consider two aspects for perioperative use of HES. First, HES can reduce intraoperative fluid loading. Recent intraoperative fluid management has changed from the liberal infusion strategy of extracellular fluid to a relatively goal-directed volume-restricted strategy using HES. Lowell et al. demonstrated that weight gain from fluid overloading during surgery correlated to high mortality rates [4]. Enhanced recovery after surgery consensus guideline [5, 6] specified “Intraoperative fluids should be balanced to avoid both hypo- and hypervolemia. Intraoperative goal directed fluid therapy should be considered on an individual basis”. In surgical use, HES (especially third-generation HES 130/0.4) demonstrated good outcomes for transfusion rate [7], mortality [8], and renal function [7]. HES still plays a major role in goal-directed intraoperative fluid therapy.

Second, HES 130/0.4 can reduce intraoperative albumin consumption because higher doses can be given compared to HES 70/0.5. Japan has not only been criticized for large albumin consumption, but also for high cost excessive perioperative albumin usage. The present study focused on this second issue.

The present study represents a large sample of 123,500 surgical patients with 10,966 patients (10,850 patients in group A and 116 patients in group B) being suitable for analysis.

Approximately 64 % of blood loss was replaced by allogeneic (52 %) and auto-transfusion (12 %). In group A, the auto-transfusion rate was 30 % of all transfusions, which was thought to be very high; however, in group B, 141 % of blood loss was replaced by allogeneic transfusion. There are two possible reasons. One reason is that a small volume of blood loss leads to hypovolemia and an unstable hemodynamic state in small children. In such situations, anesthesiologists tend to decide on early transfusion. Another reason is that the smaller the blood loss, the higher the difference between the actual blood loss and the measured blood loss because of drying gauze with the blood to be measured, and immeasurable blood absorbed by cover sheets or spilt on the floor. Anesthesiologists then tend to assess the volume status based on estimated blood loss rather than measured blood loss by the nurse.

The predicted volume of 5 and 4.4 % albumin saved was 1189 L in 1 month. This would amount to approximately 14,300 L over 1 year. Based on the statistics from the Ministry of Health, Labor and Welfare 2012 (http://www.mhlw.go.jp/stf/shingi/2r9852000002hs9l.html), the total number of surgeries performed in 1648 diagnosis procedure combination (DPC) hospitals during a 6-month period was 2,045,932, which is 2.8 times higher than the source of the present study (123,500 in 1 month). Approximately 40,000 L (14,300 L × 2.8) of albumin would be saved in a year. The consumption of albumin in Japan in 2009 was 1460,000 L (http://www.mhlw.go.jp/new-info/kobetu/iyaku/kenketsugo/2q/pdf/5-2.pdf). As a result of this analysis, 2.7 % (40,000/1,460,000) of total albumin consumption could be saved in operating theaters.

The principle proof of this analysis was recently supported by a pilot study [8] as well by a study of surgical patients [9]. Blood loss and transfusion requirements in cardiac surgery were higher for albumin compared to HES 130/0.4 [10]. Albumin administration in cardiac surgery was associated with a dose-dependent risk of acute kidney injury, whereas 6 % HES 130/0.4 was not [11]. Albumin administration for critically ill patients did not improve mortality or morbidity even for hypoalbuminemia patients [12]. Based on these findings, third-generation HES 130/0.4 may become a major colloid for perioperative volume therapy instead of albumin. In cases of massive administration of HES 130/0.4 and also of albumin, however, dilutional coagulopathy should be monitored and treated appropriately with fresh frozen plasma and platelet concentrate.

## Limitation of the study

1. As anesthesiologists may not use up to the maximum dose of HES 130/0.4 in cases of bleeding during surgery, the calculation of the possible volume of saved albumin may be overestimated.
2. The intraoperative infusion volume of 20 and 25 % albumin was excluded from the possible saving volume of albumin as it was mostly used in the priming solution of cardiopulmonary bypass circuit. This albumin, however, could also be replaced by HES 130/0.4 as the colloid for priming solution.

## Conclusion

Based on data from the 2012 JSA survey, 15,111 L of blood loss were replaced with 7970 L of allogeneic transfusion, 1777 L of auto-transfusion, 1391 L of albumin, and 7645 L of HES 70/0.5 during surgery in April 2012. The predicted volume of 5 and 4.4 % albumin saved was 1189 L (86 % of actual amount used) during this 1-month period if HES 130/0.4 had been used up to a dose of 50 mL/kg.
